# LMX1B Is Part of a Transcriptional Complex with PSPC1 and PSF

**DOI:** 10.1371/journal.pone.0053122

**Published:** 2013-01-04

**Authors:** Elisa J. Hoekstra, Simone Mesman, Willem A. de Munnik, Marten P. Smidt

**Affiliations:** 1 Department of Neuroscience and Pharmacology, Rudolf Magnus Institute of Neuroscience, University Medical Center Utrecht, Utrecht, The Netherlands; 2 Molecular Neuroscience, Swammerdam Institute for Life Sciences, University of Amsterdam, Amsterdam, The Netherlands; Radboud University, The Netherlands

## Abstract

The LIM homeodomain transcription factor *Lmx1b* is essential for the development of the isthmic organizer and mesodiencephalic dopaminergic neurons. The uncoupling of *Pitx3* and *Th* expression, in the *Lmx1b* null mutant, suggests that *Lmx1b* may act as a positional activator of the mdDA domain, eventually leading to properly differentiating mdDA neurons. In this study, we aimed to elucidate how *Lmx1b* functions mechanistically in this developmental process, by searching for molecular interactors of *Lmx1b* at the protein level. Initially, affinity-purification of LMX1B-HIS overexpressed protein in MN9D dopaminergic cells followed by mass-spectrometry analysis, resulted in the identification of PSPC1 protein as a possible binding partner of LMX1B. Subsequent immunoprecipitation experiments revealed an interaction between LMX1B and PSPC1 in a larger protein complex also containing PSF. This complex was observed *in vitro* and *in vivo*, and we hypothesize that, via PSF and PSPC1, LMX1B may be part of the previously identified *Nurr1* transcriptional complex wherein interaction with the co-repressor PSF and the transcription factor *Pitx3* is needed to drive expression of *Nurr1* target genes in specifying the dopaminergic phenotype. Furthermore, we identified GRLF1, DHX9, MYO1C, HSP70 and TMPO as potential LMX1B interactors. DHX9 and GRLF1 are highly expressed in the developing mdDA neuronal field, and GRLF1 and MYO1C have both been linked to neurite outgrowth. The identification of these proteins suggests that *Lmx1b* may act directly in the transcriptional activation of *Nurr1* target genes and be involved in other processes like neurite outgrowth as well.

## Introduction

One of the essential transcription factors involved in mesodiencephalic dopaminergic (mdDA) neuron development, is the LIM homeodomain (LIM-HD) transcription factor 1 beta (*Lmx1b*). The first sign of this relevance was provided through the analysis of the *Lmx1b* null mutant, which showed a clear midbrain defect and uncoupling of *Th* and *Pitx3* expression in the mdDA region [1]. *Lmx1b* is expressed before the expression of *Nurr1*, *Pitx3* and *Th* and has intrinsic properties as a developmental regulator. The (partial) loss of *Pitx3* and later of *Th* in the *Lmx1b* null mutant suggest that *Lmx1b* may act as an upstream activator of these genes in the development of mdDA neurons [1]–[3], or *Lmx1b* may be involved in specifying the dopaminergic niche in the midbrain region. This possibility is underlined by the fact that *Lmx1b* is also involved in regulation of *Fgf8* and *Wnt1*, and several isthmus-related transcription factors, and it is essential for inductive activity of the isthmic organizer (IsO) [4], [5]. Loss of *Lmx1b* likely affects mid-hindbrain (MHB) patterning, resulting in an early loss of a large part of the midbrain [4]. In a recent study, it was shown that specific inactivation of *Lmx1b* in mdDA progenitors, but not in the IsO, resulted in normally developing neurons, and it was suggested that *Lxm1b* is not required for the specification and differentiation of mdDA progenitors on its own [6].

Furthermore, it was shown that *Foxa1* and *Foxa2* are able to specify mdDA progenitors by positively regulating the expression of *Lmx1a* and *Lmx1b*. *Foxa1*/*Foxa2* together with *Lmx1a*/*Lmx1b*, induce expression of *Nurr1*, and it was suggested that these genes cooperate in order to promote mdDA development by regulating common targets that are important for mdDA differentiation [7]–[9].

Despite the many studies of the function of *Lmx1b* in mdDA development and differentiation, the precise role is still not clear. Most of the studies focus on identifying genes in the molecular cascades in which *Lmx1b* is involved and not much is known about the functional level of *Lmx1b* in the proposed pathways. However, two studies identified CLIM2 (LDB1) and PAX2 as proteins that directly interact with LMX1B protein, via yeast-two-hybrid assays [10], [11]. *Ldb1* is an essential LIM-HD co-factor that can, in a transcriptional complex, act as a central signaling integrator [12]–[14].

In the current study, we specifically focus on the identification of physical interactors with LMX1B protein. In an open search for binding partners, based on affinity purification, and immunoprecipitation (IP) techniques followed by mass spectrometry analysis, we identified PSPC1, GRLF1, DDX9, MYO1C, HSP70 and TMPO as possible interactors binding to LMX1B. Furthermore, via IP experiments *in vitro* and *in vivo*, a protein complex was identified containing PSPC1, PSF and LMX1B, suggesting the existence of this complex in mdDA neurons.

## Materials and Methods

### Animals

Experiments were carried out in C57Bl/6J wild-type mice (Charles River). Pregnant mice were decapitated or euthanized by CO_2_ asphyxiation and embryos were collected at E14.5 (the day on which the copulatory plug was detected was considered E0.5). Mice were maintained under standard conditions, all efforts were made to minimize suffering, and all procedures were according to and fully approved by the Dutch Ethical Committee for animal experimentation of the University Medical Center Utrecht (DEC UMC-U, The Netherlands).

### PCR and Cloning

pBSK(+)-*Lmx1b* cDNA vector (kind gift of the lab of R. Johnson, Houston) was used to clone a 1.3 kB fragment containing the full *Lmx1b* coding sequence, into the expression vector pcDNA3.1(-) (Invitrogen), by using the *Eco*RI restriction sites. To generate *myc*-HIS tagged LMX1B protein, primers were designed for the pcDNA3.1(-)-LMX1B template, to introduce an *EcoRI* restriction site at the 5′ side, and a *BamHI* restriction site at the 3′ end, plus an elimination of the stop-codon that was initially incorporated in the LMX1B construct (forward 5′-CAGAATTCGGGCGCTGGAGAGG-3′, reverse 5′-AGGATCCGGAGGCAAAGTAGGAGCTC-3′). The PCR product was cloned into pcDNA3.1(-)myc-HIS_A (Invitrogen) using the *EcoRI* and *BamHI* restriction sites. The resulting vector was sequenced (Baseclear, Netherlands).

### MN9D Cell Culture and Transfection

MN9D cells (a kind gift of Dr. Thomas Perlmann; for literature: [15], [16]) were cultured in Dulbecco’s Modified Eagle Medium (DMEM) supplemented with 10% (v/v) heat-inactivated fetal calf serum (hiFCS), 100 units/mL penicillin, 100 units/mL streptomycin and 2 mM L-Glutamine, in a standard incubator with 5% CO2 at 37°C. Cells were grown on 10 cm dishes, additionally coated with poly-L-lysine. At least 2 hours before transfection, culture medium was replaced by antibiotics free medium. Transfection was performed with Lipofectamine 2000 (Invitrogen), according to manufacturer’s protocol. Expression vector pcDNA3.1(-)-*LMX1B-*mycHIS was transfected in an amount of 22 ug DNA. Control (empty) vector was transfected in equimolar amounts. Cloning vector pBluescript SKII(+) was added as carrier DNA. 5–6 hours post transfection, the cells were split (1∶3) and cultured in fresh medium with antibiotics. Cells were harvested when the plates were 90–95% confluent and RNA or protein was isolated for further analysis.

### Ni-NTA Magnetic Agarose Bead Purification

Transfected and harvested cells were centrifuged and resuspended in Qialysis buffer (50 mM NaH2PO4, 300 mM NaCl, 10 mM imidazole, 0.05% Tween 20, pH 8.0). Lysis was done on ice for 30–40 min. and cellpellets were homogenized by passing a 27/3–4 G syringe 5 times to ensure complete cell and nuclear lysis before centrifugation. Lysates were kept on ice before being incubated with Ni-NTA magnetic agarose beads (Qiagen; 100 uL/mL beads) on an end-over-end shaker in a cold room (4–8°C) overnight. Ni-NTA beads were washed four times (50 mM NaH2PO4, 300 mM NaCl, 40 mM imidazole, 0.05% Tween 20, pH 8.0). Beads were captured using a magnetic separator (>20 mega-oersted, Qiagen). Beads were incubated in elution buffer (50 mM NaH2PO4, 300 mM NaCl, 250 mM imidazole, 0.05% Tween 20, pH 8.0) with frequent flicking of the tube, for 20 minutes. Eluates were stored for later analysis at −80°C. All buffers were supplemented with a complete protease inhibitor cocktail (Roche).

### Immunoprecipitation

MN9D or E14.5 embryonic tissue cells were homogenized in lysis buffer (50 mM Tris-HCl pH 8, 150 mM NaCl, 5 mM MgCl2, 0.5 mM EDTA, 0,2% NP-40, 5% glycerol, and 1× Protease Inhibitor (Roche)). Per plate of MN9Ds or 5–6 dissected E14.5 midbrains, approximately 500 uL lysis buffer was used. Cells were lysed for 20 minutes on ice and lysate was homogenized by passing 5× through a 27G syringe and centrifuged. Magnetic Protein A Dynal beads (Invitrogen; 35 uL beads/mL lysate) were blocked in 0,5% BSA in 1× PBS and incubated with antibody overnight on a rotator at 4°C. The following antibodies were used for (Co-)IP: rabbit anti-PSPC1 (Santa Cruz), rabbit anti-HIS (Abcam), goat anti-LMX1B (Santa Cruz), mouse anti-PSF (Sigma), and rabbit anti-NURR1 (Santa Cruz). As a control, host normal serum was used. Lysate was incubated with antibody-bound beads overnight on a rotator at 4°C. Beads were washed in lysis buffer (five times) and captured using a magnetic separator (Qiagen). Proteins were eluted in elution buffer (50 mM TrisHCl pH 8, 10 mM EDTA, 1% (v/v) SDS, and 1× Protease Inhibitor (Roche)) at 65°C for 10 minutes and with frequent vortexing. Eluates were stored for analysis at −80°C.

### Western Blot

Proteins were separated by standard SDS-PAGE on 11% acrylamide gels (Biorad), and transferred to a Hybond C extra membrane (Amersham). Membranes were blocked overnight in 5% milk powder in PBS, at 4°C. Subsequently, membranes were incubated with primary antibodies in PBS-T (0.05% v/v Tween) on a shaker for 2 hours at room temperature. Antibodies used: rabbit anti-HIS (Abcam, ab9108; 1∶20.000), rabbit anti-PSPC1 (Santa Cruz, sc-84577; 1∶10.000), goat anti-LMX1B (Santa Cruz, sc-21231; 1∶5.000), mouse anti-PSF (Sigma, B92; 1∶2.500), rabbit anti-NURR1 (Santa Cruz, sc-990; 1∶200). When using goat anti-LMX1B, blots were blocked in 5% normal donkey serum instead of milk, to reduce high background due to secondary donkey anti-goat antibody binding to bovine IgGs. Membranes were incubated with SuperSignal West Dura Extended Duration Substrate (Pierce; Thermo Scientific) and exposed to ECL films (Pierce; Thermo Scientific).

### Silverstaining

Protein eluates were separated by means of SDS PAGE. Subsequently, the gel was silver-stained to detect the proteins. The gel was fixed in 50% methanol (2×15 min) and 5% methanol (10 min), rinsed 3 times with water, soaked in 10 µM DTT (20 min), incubated in 0.1% AgNO3 (20 min), and rinsed with water. Next, it was incubated in developer solution (3% NaCO3, 0.05% formaldehyde) until protein bands were visible. The reaction was stopped by adding citric acid and the gel was washed in water. Protein bands of interest were excised form the gel and subjected to nanoLC-ESI-MS Mass Spectrometry analysis and subsequent database analysis (The Proteome Factory, Berlin). Additionally, several SDS PAGE gels were silver-stained by using the FireSilver staining kit from the Proteome Factory.

### NanoLC-ESI-MS/MS

Protein identification was performed by the Proteome Factory (Berlin, Germany): The MS system consisted of an Agilent 1100 nanoLC system (Agilent, Waldbronn, Germany), PicoTip emitter (New Objective, Woburn, USA) and a Qtof Ultima mass spectrometer (Micromass/Waters, Manchester, UK). Protein spots were in-gel digested by trypsin (Promega, Mannheim, Germany) and applied to nanoLC-ESI-MS/MS. Peptides were trapped and desalted on the enrichment column (Zorbax SB C18, 0.3×5 mm, Agilent) for five minutes using 1% acetonitrile/0.5% formic acid as eluent, then peptides were separated on a Zorbax 300 SB C18, 75 µm x 150 mm column (Agilent) using an acetonitrile/0.1% formic acid gradient from 5% to 40% acetonitrile within 40 minutes. MS spectra were automatically recorded by the mass spectrometer according to manufacturer’s instrument settings for nanoLC-ESI-MS/MS analysis. Proteins were identified using MS/MS ion search of the Mascot search engine (Matrix Science, London, England) and the non-redundant protein database (National Center for Biotechnology Information, Bethesda, USA). Ion charges in search parameters for ions from ESI-MS/MS data acquisition were set to 1+,2+ or 3+ according to the instrument’s and method’s common charge state distribution. Individual ions scores >30 indicate identity or extensive homology (p<0.05).

## Results

### Paraspeckle Protein (PSPC1) Interacts with an LMX1B-*myc*-HIS Fusion Protein

**Figure 1:**
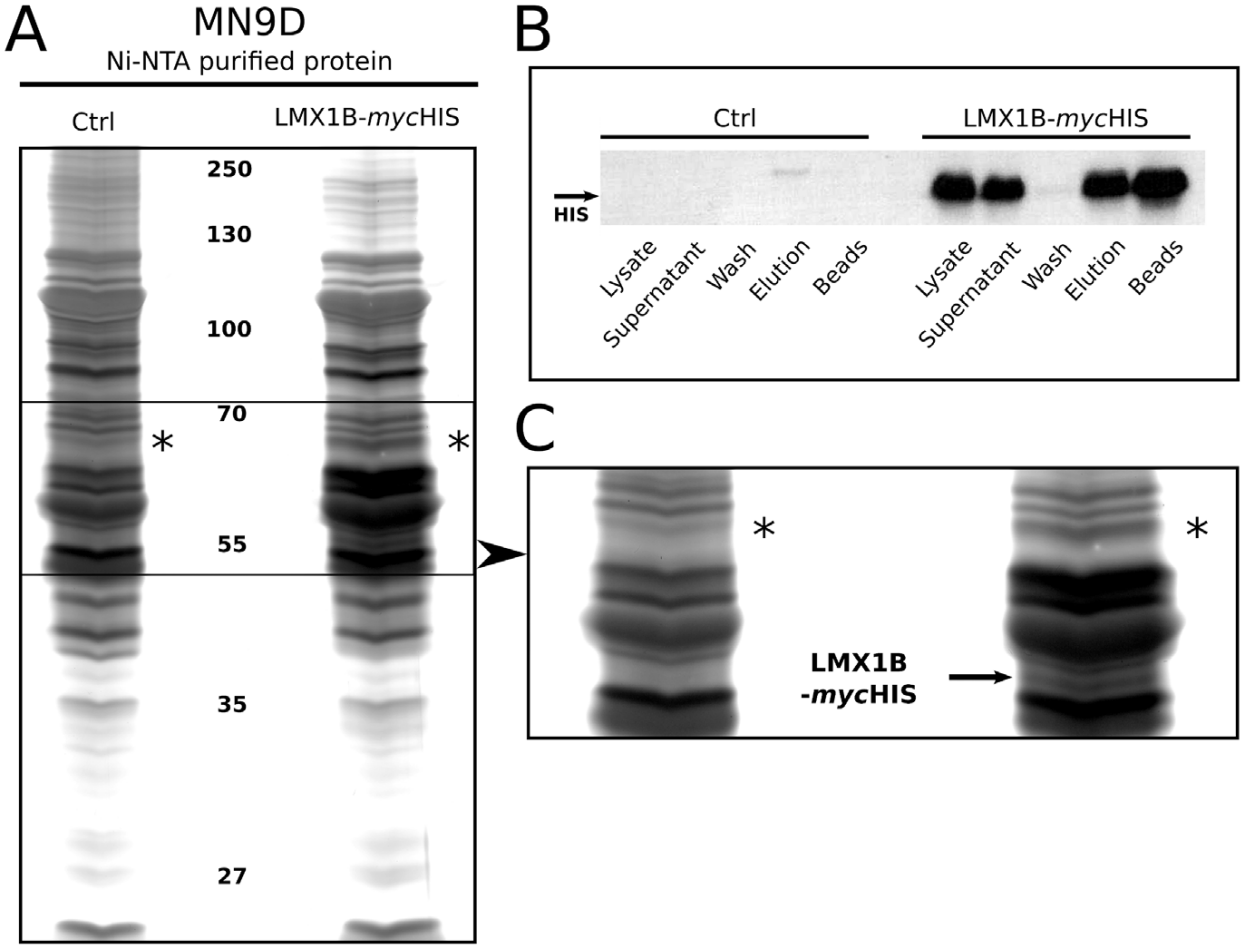
Affinity purified *Lmx1b*-HIS proteins. (A) *Lmx1b*-HIS purified proteins from MN9D cells, by means of HIS tagged affinity purification via Ni-NTA agarose beads, followed by separation on silver-stained SDS gel. The left lane represents proteins purified from control transfected MN9D cells. The right lane shows purified proteins from LMX1B-HIS overexpressing MN9D cells. Asterisks mark an observed differential protein band. (B) Western blot validation of the LMX1B-HIS overexpression in MN9D cells, followed by successful purification of LMX1B-HIS protein. Lysate shows clear LMX1B-HIS overexpression. Supernatant reveals unbound LMX1B-HIS after Ni-NTA bead incubation. Protein is strongly bound to the beads, as shown by an extremely low amount of protein that was detected in the first washing-steps. Following successful elution, beads were heated and used for a second, thorough elution; a large amount of protein was detected that was not eluted in the first elution step. (C) Detailed image of the differential protein band in the LMX1B-HIS OE sample (asterisks). Overexpressed LMX1B-HIS protein (based on size) was detected as well (arrow). *Ctrl, purified protein from control transfected MN9D cells; LMX1B-mycHIS, purified protein from MN9D cells overexpressing LMX1B-HIS.*

In order to determine possible interactors of LMX1B, an open search was performed by means of affinity purification of LMX1B. To achieve this, we overexpressed (OE) LMX1B protein translationally fused to a myc-HIS sequence, in MN9D dopaminergic cells. Through Ni-NTA affinity purification, we were able to purify the HIS-tagged LMX1B protein from cell lysates, and analyzed this by means of protein gel silver-staining (FIG. 1A). The set-up was validated by means of Western blot (FIG. 1B), that clearly revealed the successful purification of OE HIS-tagged LMX1B protein, compared to a similar purification experiment on MN9D cells transfected with an empty vector as control (pcDNA3.1(-)-myc-HIS). A large amount of HIS-tagged LMX1B protein was detected in the total lysate of OE cells, and after binding to Ni-NTA beads, unbound LMX1B-HIS remained in the supernatant. Bound LMX1B-HIS protein was retrieved after elution. Since almost no protein was detected in the first washing steps, the LMX1B-HIS protein was strongly bound to the Ni-NTA beads.

Due to the large amount of background protein, only small differences were observed in protein composition of both samples. In the OE sample lane, a small, extra protein band was observed at approximately 55 kDA, which most likely represents the LMX1B-HIS OE protein, based on the size. In addition, a clear protein band was observed between 65 and 70 kDA (FIG. 1A and C, asterisks). This protein band was excised from gel, together with the same material (same relative mobility position) from the control lane, and sent for mass spectrometry analysis (Proteome Factory, Berlin). The resulting data analysis (MASCOT) identified several proteins (Table 1). Unfortunately, three of the four identified proteins, were also found in the control sample, and they might represent background proteins. However, one protein was identified which was not present in the control lane: Paraspeckle protein 1 (PSPC1).

**Table 1:** Mascot search results of proteins from the Ni-NTA purification set-up.

Protein name	Uniprot name	Mass	Score	Peptide match	In control protein band
Heterogeneous nuclear ribonucleoprotein L	HnRNP L	60085	193	6	yes
**Paraspeckle protein 1**	**PSPc1**	**58736**	**138**	**4**	**no**
DEAD (Ala-Glu-Ala-Asp) box polypeptide 5	Ddx5	69237	108	2	yes
Splicing factor 1	Sf1	63100	90	3	yes

### *Pspc1* is Expressed in the mdDA Neuronal Field, Overlapping with *Lmx1b* Expression

**Figure 2:**
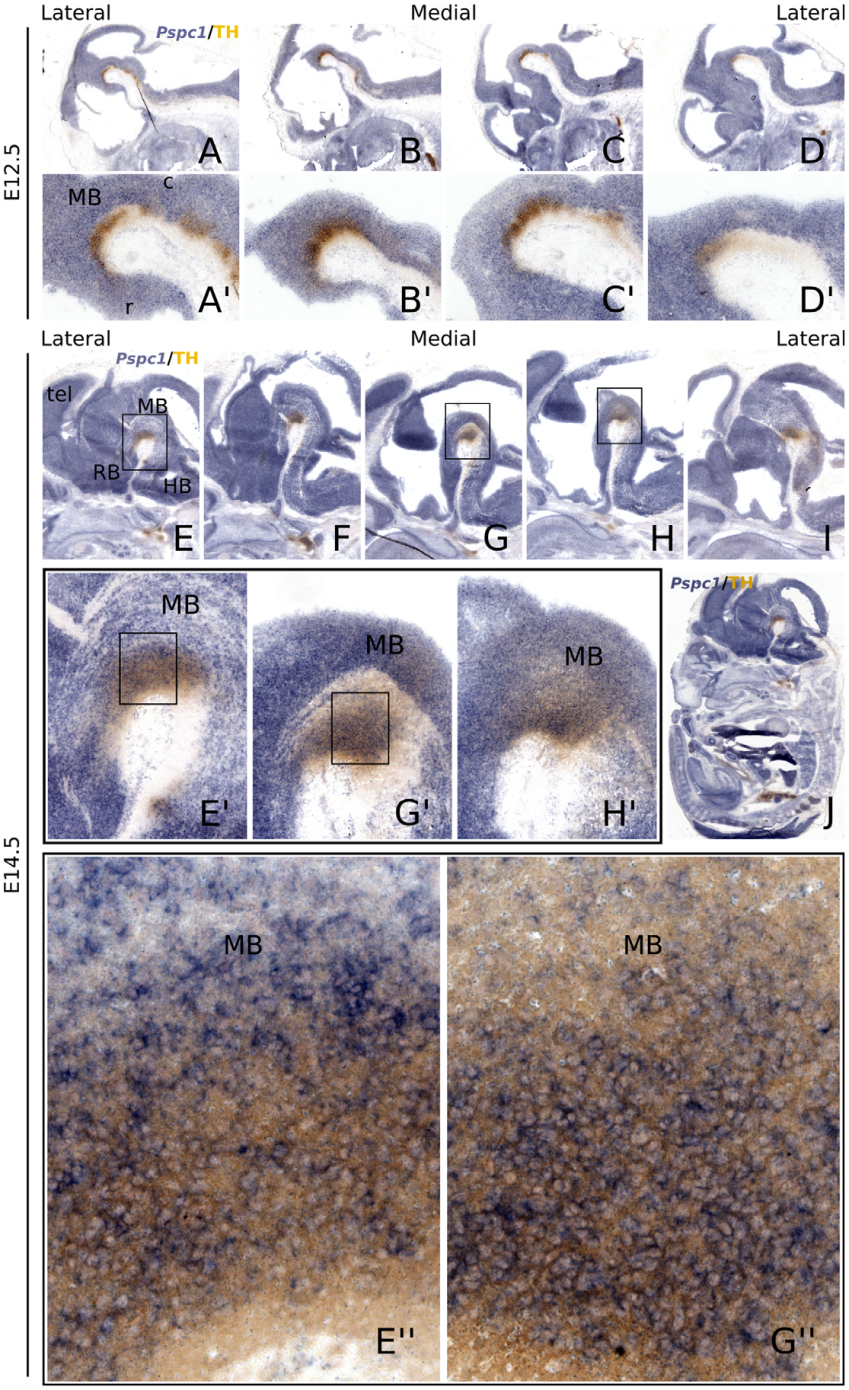
*Pspc1* is expressed in the midbrain during development. (A-D’) Sagittal analysis of the expression pattern of *Pspc1* transcript (blue) in E12.5 wild-type (C57Bl/6J) mice. TH immunohistochemistry staining (brown) was taken along to mark the mdDA neuronal field. (E-I) At E14.5, *Pspc1* is expressed in the mdDA area, but also in the rest of the CNS, as revealed by an overview image (J). (E’-H’) High levels of Pspc1 transcript are observed in the brain, overlapping with the TH positive domain. (E”-G”) Pspc1 is co-expressed in most TH positive neurons.

To address whether the described interaction between LMX1B-HIS and PSPC1 is relevant in mdDA neurons, we performed in situ hybridization (ISH) analysis of *Pspc1* on E12.5 and E14.5 wild-type (C57Bl/6J) tissue, with TH immunohistochemistry as a reference. At E12.5, *Pspc1* transcript was observed throughout the entire CNS (FIG. 2A–D), and was clearly present in the mdDA area (FIG. 2A’-D’). At E14.5, a similar expression pattern was detected; *Pspc1* was broadly but specifically expressed in several embryonic areas and CNS (FIG. 2E–I, and J), thereby clearly overlapping with the TH expression domain (Fig. 2.E’–H’). When analyzing the mdDA neuronal field into more detail, *Pspc1* mRNA was expressed in TH positive neurons in the mdDA neuronal field (FIG. 2E”,G”).

To conclude, *Pspc1* transcripts were clearly present in the developing midbrain and mdDA neurons, suggesting that the found interaction may be relevant in terms of *Lmx1b* function in mdDA neurons.

### PSPC1 is not Present in LMX1B Immunoprecipitated Complexes

**Figure 3:**
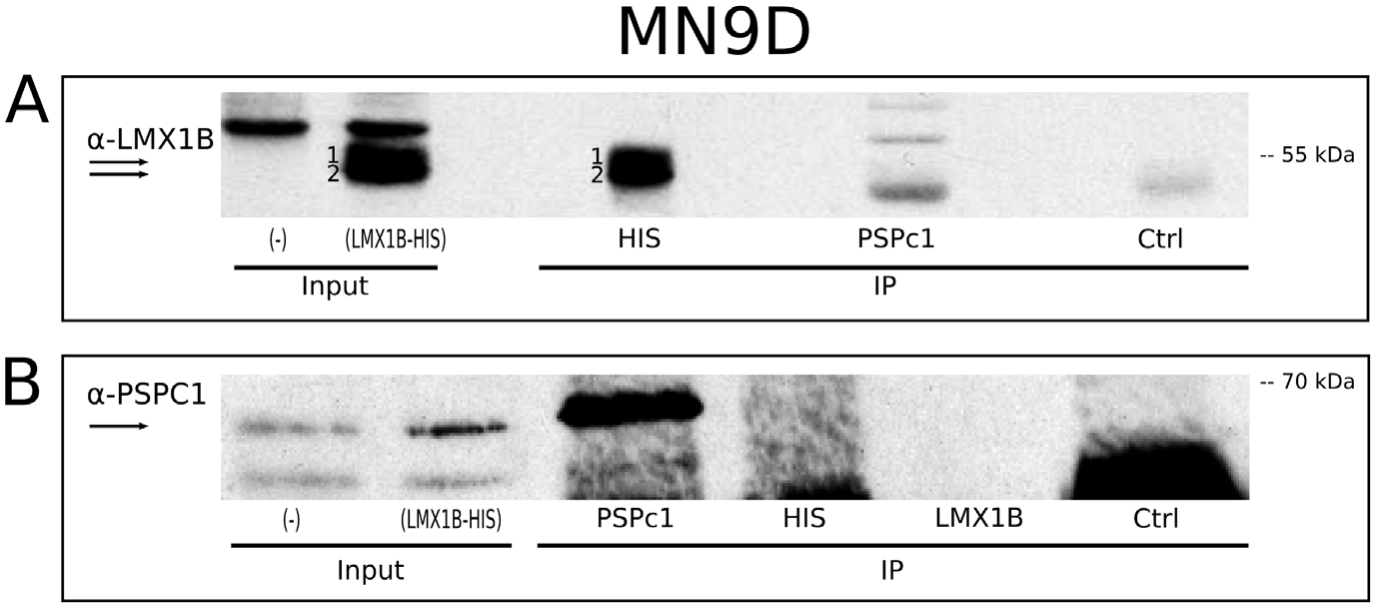
Pull-down of LMX1B(-HIS) and PSPC1 protein from MN9D cells. (A) Overexpression of LMX1B-HIS reveals two specific protein bands (1,2). HIS IP shows clear protein bands (1,2). PSPC1 IP reveals three bands of which none completely overlaps with the HIS IP protein bands. (B) PSPC1 is clearly pulled down in a PSPC1 IP. No PSPC1 interaction could be detected in the HIS IP. *HC, heavy chain detection; Ctrl, control IP with normal host serum; IP, immunoprecipitation; (-), MN9D cells transfected with control (empty) vector; (OE) MN9D cells transfected with LMX1B-HIS overexpression vector.*

Since the outcome of the mass spectrometry analysis and the overlapping transcripts of *Lmx1b* and *Pspc1* in the developing midbrain, suggested a potential interaction, we aimed to validate this by immunoprecipitation (IP). In order to achieve this, we transfected MN9D cells with LMX1B-HIS and control constructs and IP experiments were performed with antibodies against LMX1B, HIS, and PSPC1 (FIG. 3).

Western blot analysis with an LMX1B antibody clearly showed successful overexpression and HIS-immunoprecipitation of LMX1B-HIS protein (FIG. 3A, bands 1,2). In the PSPC1 IP, several protein bands were detected (FIG. 3A), however the observed protein of interest appeared to be located in a position that runs lower than the detected LMX1B-HIS protein bands (1 and 2). Moreover, in this experiment, the product observed in the control IP, clearly suggested partial overlap with the product in the PSPC1 IP. Furthermore, the performed PSPC1, LMX1B and HIS IP experiments were analyzed on Western blot by anti-PSPC1 antibody. We confirmed PSPC1 pull down in the PSPC1 IP, but we were not able to detect PSPC1 protein in either the HIS IP, or LMX1B IP at the expected size (FIG. 3B).

### LMX1B Interacts with the Previously Identified Binding Partner of PITX3 and NURR1, PSF

The previously described interaction between PSPC1 and PSF [17]–[19], and the fact that PSF was coupled to a recently described NURR1/PITX3 transcriptional protein complex [20], made us hypothesize that LMX1B may also interact with PSF. Therefore, we aimed to investigate a possible interaction between LMX1B and PSF through co-immunoprecipitation experiments.

**Figure 4:**
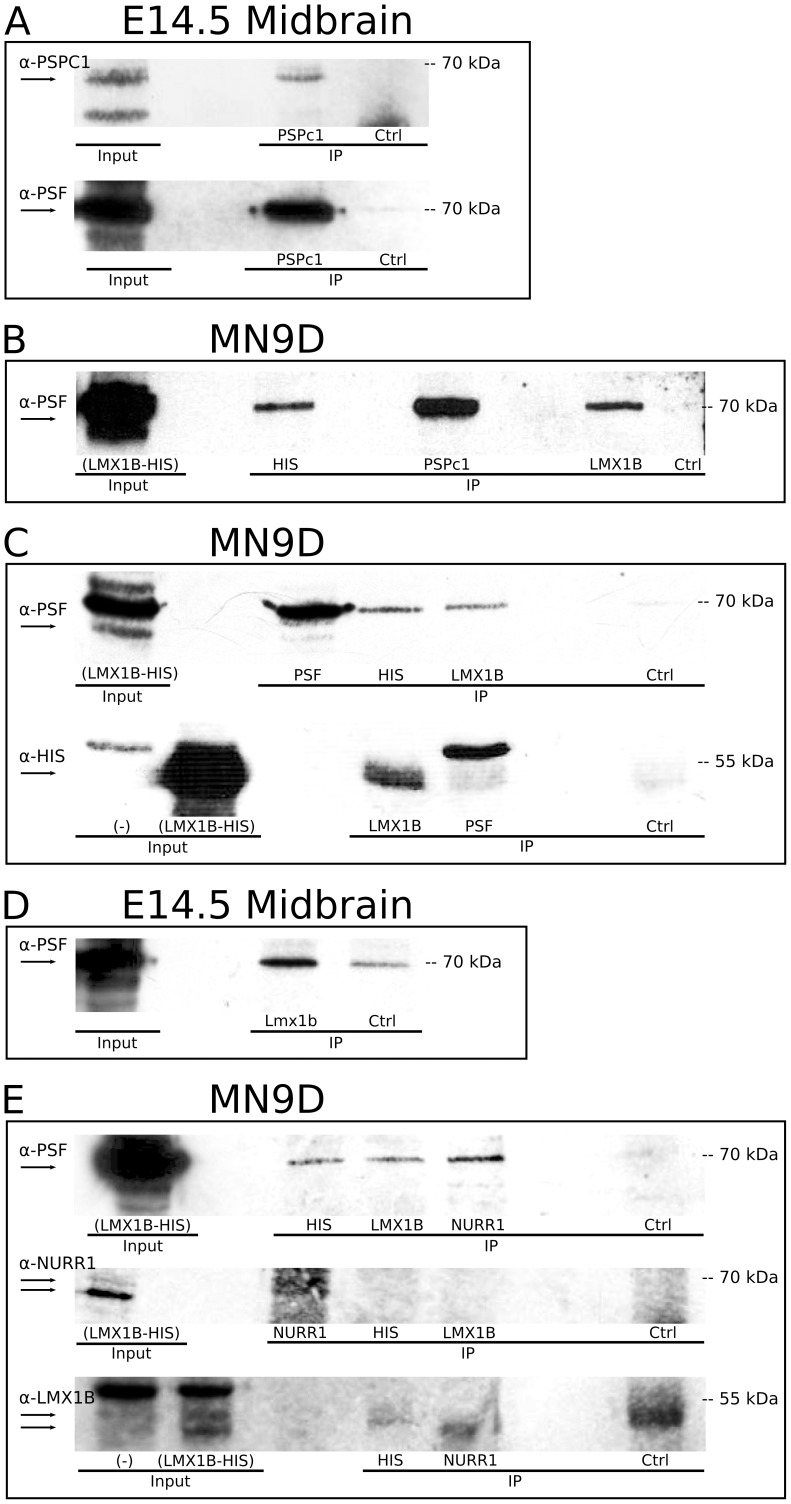
Possible interaction between PSPC1 and PSF, and between PSF and LMX1B. (A) PSPC1 is detected in E14.5 midbrain neurons, and has an interaction with PSF in these cells. (B) An interaction with PSF is shown in HIS IP, PSPC1 IP and LMX1B IP, in MN9D cells overexpressing LMX1B-HIS. (C ) Again, PSF interacts with LMX1B, as shown in a HIS and LMX1B IP. LMX1B IP reveals clear pull-down of LMX1B-HIS, which also observed in the PSF IP, and in lower amount in the control IP. (D) PSF interacts with LMX1B in developing midbrain neurons. (E) Confirmation of the interaction of PSF with LMX1B(-HIS), and with NURR1. NURR1 IP reveals pull down of the protein, however this is not shown in HIS or LMX1B IP. Moreover, in a NURR1 IP, no LMX1B could be observed. *Ctrl, control IP with normal host serum; IP, immunoprecipitation; (-), MN9D cells transfected with control(empty) vector; (LMX1B-HIS) MN9D cells transfected with LMX1B-HIS overexpression vector.*

We first confirmed the described interaction between PSPC1 and PSF, *in vitro* and *in vivo*. IP experiments with anti-PSPC1 were performed on total lysate from MN9D cells, and E14.5 dissected midbrain material. Western blot analysis revealed successful pull down of endogenous PSPC1, and more importantly, we validated that PSF indeed co-immunoprecipitated with PSPC1, suggesting a clear physical interaction under these conditions (FIG. 4A). Similar IP experiments on MN9D cell lysates confirmed the PSF/PSPC1 interaction (FIG. 4B).

As we have demonstrated that PSF interacts with PSPC1, we additionally aimed to test the possible interaction of LMX1B with PSF. Importantly, HIS and LMX1B IP experiments on MN9D cell lysates showed a clear interaction with PSF (FIG. 4B,C). In addition, when performing an IP against PSF, LMX1B-HIS protein could be detected (FIG. 4C). To determine whether the interaction of LMX1B with PSF also exists *in vivo*, IP was performed on E14.5 dissected midbrain material (FIG. 4D). In agreement with the observed interaction *in vitro*, we could clearly detect PSF protein after pull-down of LMX1B (FIG. 4D), thereby confirming that this interaction also exists in the developing midbrain.

As mentioned above, in a previous study, a *Nurr1* transcriptional complex was identified in which PITX3 and NURR1 showed an interaction with PSF [20]. Since we found interactions between LMX1B and PSF, in cells and in E14.5 midbrain tissue, we hypothesized that LMX1B might be involved in this transcriptional complex as well. We therefore analyzed a possible direct interaction between LMX1B and NURR1. IP was performed on LMX1B-HIS OE MN9D cell lysate, with pull-down of NURR1, HIS and LMX1B (FIG. 4E). We were able to confirm the binding of PSF to all three immunoprecipitated proteins. However, the direct interaction with NURR1 was not detected in HIS or LMX1B IP experiments. After pull-down of HIS-tagged protein, two LMX1B-HIS products were observed, however in the NURR1 IP, these bands were absent (FIG. 4E). Altogether, despite the identified interactions of LMX1B with PSF, and PSF with NURR1, we were not able to confirm direct binding of LMX1B with NURR1. It is still possible that LMX1B is involved in the *Nurr1* transcriptional complex, but likely via indirect interaction through PSF.

### Identification of a 230 kDa Protein Complex Containing PSPC1, PSF and LMX1B

**Figure 5:**
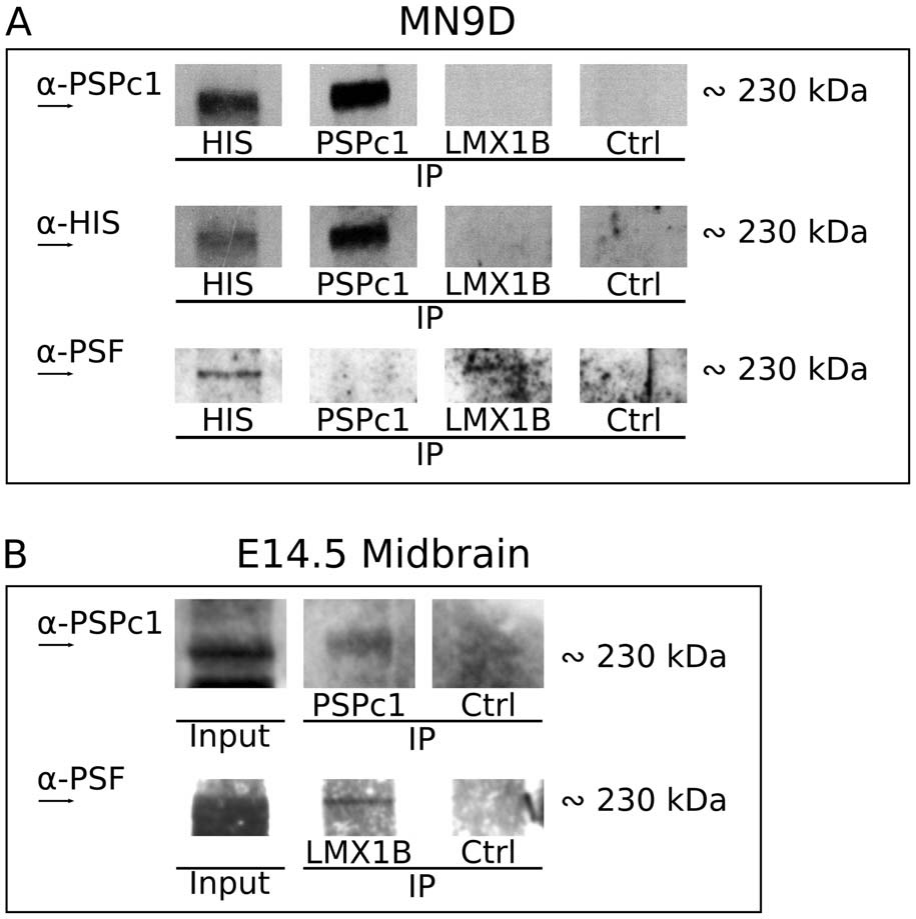
Identification of a 230 kDa complex containing LMX1B(-HIS), PSPC1 and PSF. (A) PSPC1 protein is identified in a protein band of approximately 230 kDa, in a PSPC1 IP and in a HIS IP, but not in an LMX1B IP. When immunoblotting for HIS, a protein of the same size is detected in these IP experiments, and again not in an LMX1B IP. After immunoblotting for PSF, of the same blot, PSF is detected at 230 kDa in the HIS IP, faintly in the PSPC1 IP, and in the LMX1B IP. (B) Analysis of 230 kDa proteins *in vivo*, in E14.5 dissected midbrain tissue. PSPC1 pull-down reveals PSPC1 protein at 230 kDa, suggesting that the 230 kDa protein complex exist *in vivo*. At the same height, PSF is detected when immunoprecipitating for LMX1B. *Ctrl, control IP with normal host serum; IP, immunoprecipitation.*

In contrast to the absence of PSPC1 protein in LMX1B-(HIS) IP experiments, at the expected size, a clear protein band was observed at approximately 230 kDa (FIG. 5A). At this position, a protein band was observed after Western analysis with a PSPC1 antibody and an LMX1B antibody in HIS IP experiments and PSPC1 IP experiments, respectively (FIG. 5A). This suggests that the hypothesized interaction between PSPC1 and LMX1B-(HIS) might exist in a larger complex, that is detected at 230 kDa. Surprisingly, at the 230 kDa position, also PSF was detected in a HIS IP, and in an LMX1B IP. However, we were not able to detect this PSF protein at 230 kDa in a PSPC1 IP (FIG. 5A). The presence of these proteins at 230 kDa, make it tempting to hypothesize that one or more protein complexes exists, containing PSPC1 (70–65 kDa), PSF (100, 95 and 75 kDA) and LMX1B-(HIS) (55–50 kDa), and that this complex is strong enough to remain intact in Western analysis. Since this result might suggest an alternative complex of LMX1B, PSPC1 and PSF we aimed to validate this complex *in vivo*. E14.5 dissected midbrains were used for IP of PSPC1 and LMX1B. Western analysis demonstrated a specific PSPC1 positive signal at 230 kDa, indicating that we could pull-down this PSPC1-containing protein complex, *in vivo* (FIG. 5B). Interestingly, also the presence of PSF at 230 kDa was confirmed, in LMX1B IP experiments (FIG. 5B).

To conclude, PSF and PSPC1, together with LMX1B(-HIS) were identified through IP experiments in a protein complex of 230 kDa, *in vitro* and *in vivo*.

### LMX1B Interacts with TMPO and HSP70

**Figure 6:**
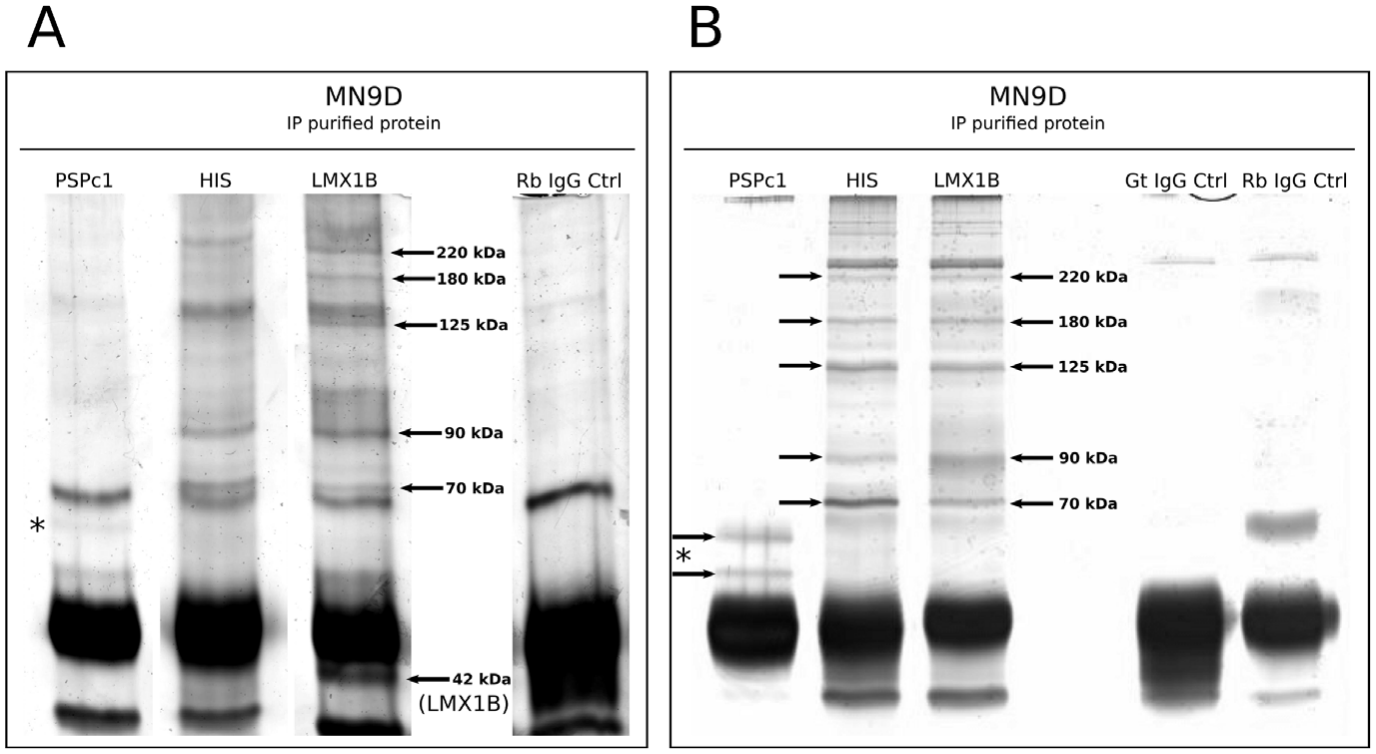
LMX1B co-immunoprecipitated protein identification. (A) IP against HIS, PSPC1 and LMX1B on LMX1B-HIS OE MN9D cell lysate, followed by separation on silver-stained SDS gel. The PSPC1 IP shows two protein bands that might represent both PSPC1 isoforms. Five protein bands of the LMX1B IP were excised and used for mass spectrometry analysis (arrows). In addition, the 42 kDa protein was taken along, as a positive control, and was later confirmed as LMX1B protein. (B) Second IP experiment against HIS, PSPC1 and LMX1B on LMX1B-HIS OE MN9D cell lysate, followed by separation on silver-stained (FireSilver kit, Proteome Factory, Berlin) SDS gel, confirming and improving the pattern as described in the first IP experiment (A). The PSPC1 IP shows two protein bands that likely represent both PSPC1 isoforms, and they were used for mass spectrometry analysis. Five differential protein bands of the LMX1B and HIS IP were excised and used mass spectrometry analysis (arrows). *Gt IgG Ctrl, goat IgG control; Rb IgG Ctrl, rabbit IgG control.*

In order to further examine other possible interactions of unknown proteins with LMX1B we used a different purification set-up based on IP experiments with antibodies against PSPC1, HIS and LMX1B and analyzed this on silver-stained gels (FIG. 6). Compared to the initially used Ni-NTA purification method, the IP experiments yielded very clean results in terms of protein background. PSPC1 IP resulted in two clear protein bands that were absent in the control IP (FIG. 6, asterisks). Since they range between 60 and 65 kDa, they may represent two PSPC1 protein isoforms, that were detected before on Western blot. Most interestingly, HIS and LMX1B IP samples display protein separation patterns that were almost identical between the different IP samples. Several protein bands were clearly and specifically observed in all IP experiments and were absent in the control IP. To further analyze some of these proteins, all promising bands were excised and subjected to mass spectrometry analysis (The Proteome Factory, Berlin) (FIG. 6A,B, arrows). The Mascot results of this analysis are shown in table 2, 3 and 4.

**Table 2:** Mascot search results of proteins from LMX1B IP purified excised protein bands.

Excised fragment	Protein name	Uniprot name	Mass	Score	Peptide match
**220 kDa**	Keratin 15	KRT15	49086	70	3
	Dystrophia myotonica-containing WD repeat motif	DMWD	65572	23	1
**180 kDa**	Keratin 15	KRT15	49086	59	1
	Keratin 75	KRT75	42357	46	2
**125 kDa**	**Myosin 1c**	**MYO1C**	**118082**	**113**	**6**
**95 kDa**	**Lamina-associated polypeptide 2, isoforms alpha/zeta**	**LAP2/TMPO**	**75285**	**169**	**5**
	Proto-oncogene protein C-ros	ROS1	10510	40	1
	FERM and PDZ domain-containing protein 1	FRMPD1	169102	40	1
	Cyclic AMP specific phosphodiesterase	PDE4D5A	23890	33	1
**70 kDa**	Keratin 8	KRT8/Card2	54514	104	1
	**Heat shock protein 70**	**HSP70**	**70793**	**51**	**3**
	X-ray repair complementing defective repair in Chinese hamster cells 6	KU70/Xrcc6	69442	26	3
**42 kDa**	**LIM homeobox transcription factor 1 beta**	**LMX1B**	**41541**	**81**	**5**
	Actin related protein 3	ACTR3	47327	67	3
	Histocompatibility 2, class II antigen E beta 2	H2-EB2	29003	19	1
	Integrin beta-4	ITGB4	101086	19	1

**Table 3:** Mascot search results of proteins from HIS IP purified excised protein bands.

Excised fragment	Protein name	Uniprot name	Mass	Score	Peptide match
**220 kDa**	Analysis failed	–	–	–	–
**180 kDa**	Titin isoform N2-A	TTN	3713700	60	26
	**Glucocorticoid receptor DNA-binding factor 1**	**GRLF1**	**170285**	**52**	**5**
	mCG142711	mCG142711	76465	30	7
**125 kDa**	Titin isoform N2-A	TTN	3713700	41	18
	Msx2-interactin protein	SPEN	398292	32	4
**90 kDa**	Titin isoform N2-A	TTN	3713700	35	17
	Cardiomyopathy-associated protein 5	CMYA5	412787	32	7
**70 kDa**	Axonemal dynein heavy chain	DNAHC	474513	31	7
**65 kDa**	Titin	TTN	3764005	38	18
	Coiled-coil domain-containing 15	CCDC15	94848	31	2
**60 kDa**	Leucine-rich repeat-containing protein 16b	LRRC16B	150322	37	2
	Family with sequence similarity 186, member A	FAM186A	194430	31	3

**Table 4:** Mascot search results of proteins from LMX1B-2 IP purified excised protein bands.

Excised fragment	Protein name	Uniprot name	Mass	Score	Peptide match
**220 kDa**	Analysis failed	–	–	–	–
**180 kDa**	**DEAH (Asp-Glu-Ala-His) box polypeptide 9**	**DHX9/DDX9**	**131636**	**70**	**5**
**125 kDa**	Keratin 8	KRT8	54514	38	1
**90 kDa**	**Lamina-associated polypeptide 2 isoform alpha**	**LAP2/TMPO**	**75122**	**334**	**13**
**70 kDa**	**Heat shock protein 70**	**HSP70**	**70793**	**175**	**4**

As mentioned above, we hypothesized that the 60 and 65 kDa bands might represent PSPC1 protein. Yet, mass spectrometry analysis failed to confirm this. The proteins identified in these bands yielded very low scores, each with a value of approximately 30 and only two peptide queries matched per sample (data not shown). Therefore the identified proteins likely were background proteins or false hits.

Of the HIS IP experiment, five protein bands were analyzed (FIG. 6B,arrows and Table 3). Unfortunately, the 220 kDa protein analyzes failed, and furthermore, several other protein bands resulted in the identification of Titin protein (Table 3). This might be a contamination of the sample. Nonetheless, the unique peptide numbers matched, were extremely high, and from literature, it is known that Titin, which is the largest protein currently known, has at least one polyhistidine stretch in one of its domains [21], [22]. Likely, the IP with anti-HIS antibody resulted in the pull-down of (part of) this Titin protein, due to high affinity for the poly-histidine stretch. Therefore, in this study, we only focused on the other proteins identified. One protein displayed both a relatively high protein score (52), and high number of peptide queries matched (5): Glucocorticoid receptor DNA-binding factor 1 (GRLF1) (Table 3; 180 kDa protein band). Based on the number of unique peptide matches, mCG142711, Msx2-interacting protein (SPEN), cardiomyopathy-associated protein 5 (CMYA5) and axonemal dynein heavy chain (DNAHC), are interesting as well. Notably, these two identified proteins do not match in theoretical size compared to the position in the silver stained gel. However, the Mascot-data indicated that these proteins are significant hits.

Importantly, of the two separate LMX1B IP experiments, five protein bands were analyzed of each IP. Additionally, as a control, we also sent a 42 kDa protein band, suspected to be LMX1B, for analysis, and this was indeed confirmed with 5 peptide queries matched and a relatively high score of 81 (FIG. 6A and Table 2, 42 kDa band). The two separate LMX1B IP experiments (FIG. 6A,B, arrows) resulted in the mass spectrometry identification of several highly interesting proteins. Based on the combination of 6 unique peptide-queries matched and a relatively high score of 113, Myosin 1c (MYO1C) can be considered as a promising potential binding partner of LMX1B (Table 2; 125 kDa sample). The second LMX1B IP resulted in the identification of DEAH (Asp-Glu-Ala-His) box polypeptide 9 (DHX9/DDX9). The Mascot analysis revealed a number of 5 peptide-queries matched and a high score. However, the true size of this protein is 132 kDa whilst it was identified in the 180 kDa sample (Table 4). Intriguingly, when comparing the two LMX1B IP experiments, two protein hits were found in both (Table 2 and 4). Heat shock protein 70 (HSP70) was identified in the two separate 70 kDa protein bands, with peptide-queries matched of respectively 3 and 4, which is lower than the threshold of 5 that we were applying for the selection of the other proteins. However, the individual scores are high and the fact that this protein is identified in two different samples makes it very likely that this protein is a potential interactor of LMX1B. Finally, the most interesting protein that was identified, in both 95 kDa samples, is Lamina-associated polypeptide 2 (LAP2/TMPO). In both IP experiments, it displayed the highest scores among all significant proteins identified, and moreover, in the second LMX1B IP, 13 unique peptide-queries were matching with TMPO.

Taken together, several proteins were identified with a Mascot score above a cut-off of 50, and with a peptide-queries match of preferably 5 or higher: GRLF1, DHX9 and MYO1C. The two novel proteins HSP70 and TMPO were identified in two separate experiments and may therefore represent the most reliable results.

## Discussion

**Figure 7:**
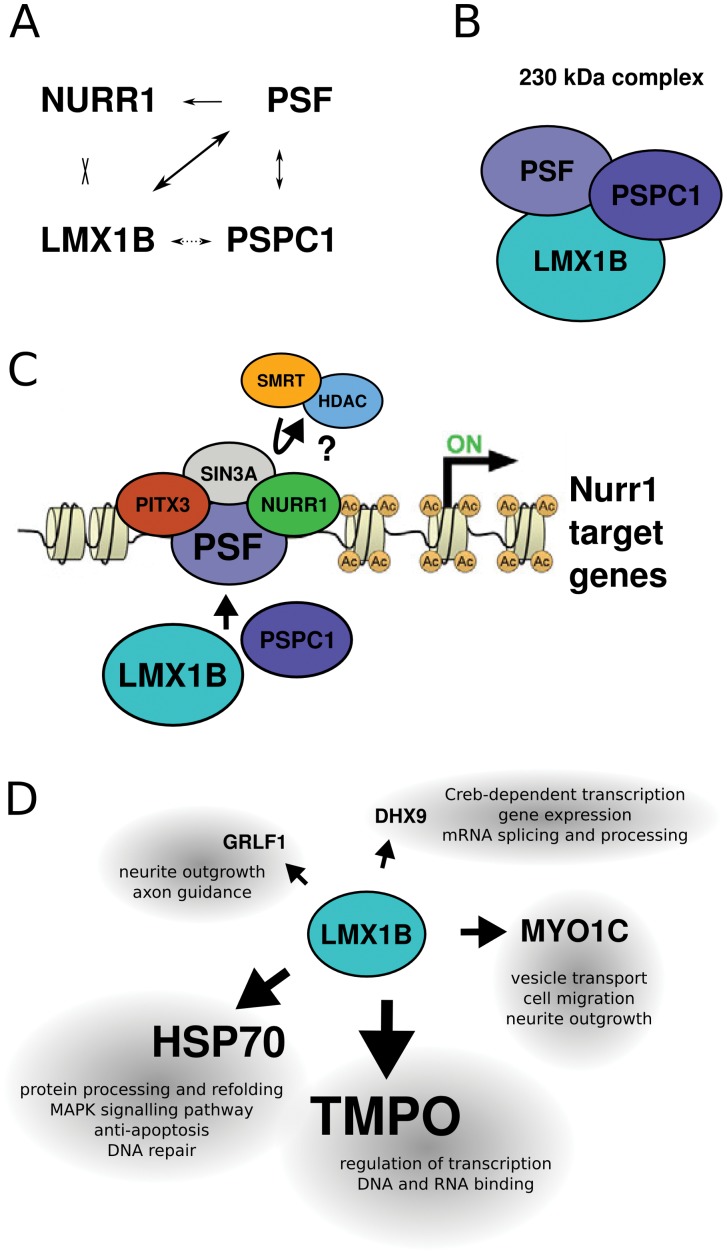
Schematic representation of suggested protein interactions with LMX1B. (A) Interactions were identified by means of immunoprecipitation experiments. Direct interactions between NURR1 and PSF, between PSF and LMX1B and between PSF and PSPC1 were found. (LMX1B)-HIS interaction with PSPC1 was identified in a 230 kDa complex only. (B) Western analysis revealed interactions between PSF, PSPC1 and LMX1B at 230 kDa, suggesting a complex of this size containing all three proteins. (C) The *Nurr1* transcriptional complex (adapted from [20]. We hypothesize that LMX1B might have a function in this complex as well, by physically binding to PSF, maybe via PSPC1. (D) Novel direct interactors of LMX1B identified in a screen in which IP experiments were analyzed on silver-stained protein gels and by mass-spectrometry analysis.

To acquire a better understanding of the functional role of *Lmx1b* in mdDA development, we aimed to identify proteins that have a physical interaction with LMX1B, and we initiated this with an open screen for direct interactors. HIS-tagged affinity purification of LMX1B protein, followed by mass spectrometry resulted in the identification of Paraspeckle protein PSPC1. However, by means of IP, we initially could not confirm an interaction between LMX1B and PSPC1. Importantly, a 230 kDa protein complex was identified in several IP experiments, that contained PSPC1, PSF and LMX1B (FIG. 7B). So, despite the lack of a confirmed single interaction between LMX1B and PSPC1, we were able to confirm an interaction between these proteins in a larger complex, also containing PSF, a known interactor of PSPC1 and confirmed here. PSPC1 belongs to the family of DBHS (Drosophila behavior and human splicing) proteins, that have several conserved domains in common, of which one is the NONO/Paraspeckle (NOPS) domain [23]. Within this highly conserved DBHS domain, the three DBHS proteins PSPC1, NONO and PSF share 70% sequence identity, and the domain is essential for homodimerization and heterodimerization of the three proteins with each other [17], [19]. Recently, PSF was identified as a crucial co-factor in a *Nurr1* transcriptional complex, during mdDA development [20]. The finding of PSPC1 in a 230 kDa complex with LMX1B and PSF made us hypothesize that, to perform transcriptional functions in mdDA development, LMX1B might be part of this *Nurr1* transcriptional complex, via PSPC1 and PSF (FIG. 7C). Importantly, in this study we validated that LMX1B and PSF have a physical interaction, in the identified larger protein complex, but also at the single protein level. To further address a possible interaction of LMX1B in this *Nurr1* transcriptional complex, we investigated a potential, direct interaction between LMX1B and NURR1, but were unable to confirm this (FIG. 7A). However, it is still possible that LMX1B is involved in the transcriptional complex, but not through direct interaction with NURR1.

### Novel Potential LMX1B Interacting Proteins

In addition to the screen via affinity purification, a second set-up was used to find novel interacting proteins of LMX1B (FIG. 7D). For this, IP purification of LMX1B, HIS and PSPC1 protein was performed, followed by SDS-PAGE, silver-staining and mass spectrometry analysis. The clear confirmation of LMX1B, which was taken along as a positive control, indicates that the set-up is valid for purifying proteins and identifying them by means of mass spectrometry.

A 125 kDa protein band resulted in the identification of Myosin 1c protein (MYO1C), that has a known size 118 kDa. Mass spectrometry analysis showed that 6 different peptide-queries all matched with MYO1C, and also the Mascot score was relatively high, strongly indicating that this identified protein is a true hit. The Myosin protein 1 family consist of actin-based molecular motors, which have various functions in vesicle transport, and docking to the plasma membrane [24], [25]. The protein is expressed ubiquitously [26], and expression in the brain is mainly found in neurons [25]. Based on online expression databases, *Myo1c* mRNA is found throughout the brain with enriched expressions in the dorsal midbrain, in the developing midbrain oculomotor complex region. A more general function of *Myo1c* is cargo delivery and membrane trafficking, and the protein has been linked to cell migration and neurite outgrowth [25], [26], making it a promising protein for further investigation of its role in binding to LMX1B, and a possible function in mdDA development.

In addition, we identified Glucocorticoid receptor DNA-binding factor 1 (GRLF1; 170 kDa), and DEAH (Asp-Glu-Ala-His) box polypeptide 9 (DHX9/DDX9/RHA; 132 kDA) which is smaller than the expected 180 kDa based on the observed separation on gel (silver stain). DHX9, also known as RNA helicase A (RHA), has a role in Creb-dependent transcription, but has additionally been implicated in nuclear export of unspliced viral RNA’s [27]. DEAH helicase domains, like homeobox domains, are involved in ATP-DEPENDENT chromatin remodeling or bind DNA and post-translationally modified nucleosomes, in either way influencing gene expression [28]. GRLF1 (p190RhoGAP) functions in neurite outgrowth [29] and mice lacking GRLF1 show defects in axon guidance and fasciculation [30]. Furthermore, GRLF1 interacts with plexins, and is essential in semaphorin signaling to the actin cytoskeleton [31]. Both genes show high mRNA levels in the developing brain and are clearly expressed in the developing midbrain, and in the adult mdDA system (online expression databases: Genepaint.org and the Allen Brain Atlas).

Finally, two proteins were identified in two different IP experiments simultaneously. Heat-shock protein 70 (HSP70/HSPA1B), was identified from 70 kDa protein bands. The fact that this protein is identified twice in separate experiments, enhances the possibility that this represents a true Lmx1b interacting protein. It is a chaperone protein that is highly expressed in response to cell stress [32]. The protein has been implicated in brain development and prevents protein misfolding and aggregation associated with neurodegenerative diseases like Alzheimer’s disease and Parkinson’s disease [32]. That study also showed that administration of this protein to mdDA neurons can rescue the cells from apoptosis.

The other potential interactor, Lamina-associated polypeptide 2 (LAP2/TMPO), was identified in the 90 kDa protein bands of the two separate LMX1B IP experiments. In both mass spectrometry analyzes, a high peptide-queries match and score was found. *Lap2/Tmpo* encodes seven mouse thymopoietin (TMPO) mRNA transcripts, that encode ubiquitously expressed nuclear proteins [33]. Not much is known about the exact function of the protein and no literature about any role in neuronal development was found. The gene is expressed in the developing brain, but in low levels. However, it is expressed in higher levels in the ventricular zone, partially overlapping with the most caudal dorsal mdDA neuronal field (online expression databases).

The double-independent identification of the above described binding partners of LMX1B, under the described conditions, suggest that these proteins are able to interact with LMX1B and modify its functional aspects. The exact role of these proteins in *Lmx1b* function awaits analysis through *in vivo* manipulation, determining which part of the *Lmx1b* phenotype may be depending on a specific LMX1B protein-protein interaction event.
